# Strain-Modulated Magnetism in MoS_2_

**DOI:** 10.3390/nano12111929

**Published:** 2022-06-04

**Authors:** Hongtao Ren, Gang Xiang

**Affiliations:** 1 School of Materials Science and Engineering, Liaocheng University, Hunan Road No. 1, Liaocheng 252000, China; 2 College of Physics, Sichuan University, Wangjiang Road No. 29, Chengdu 610064, China

**Keywords:** straintronics, spintronics, web buckles, thickness-dependence

## Abstract

Since the experiments found that two-dimensional (2D) materials such as single-layer MoS_2_ can withstand up to 20% strain, strain-modulated magnetism has gradually become an emerging research field. However, applying strain alone is difficult to modulate the magnetism of single-layer pristine MoS_2_, but applying strain combined with other tuning techniques such as introducing defects makes it easier to produce and alter the magnetism in MoS_2_. Here, we summarize the recent progress of strain-dependent magnetism in MoS_2_. First, we review the progress in theoretical study. Then, we compare the experimental methods of applying strain and their effects on magnetism. Specifically, we emphasize the roles played by web buckles, which induce biaxial tensile strain conveniently. Despite some progress, the study of strain-dependent MoS_2_ magnetism is still in its infancy, and a few potential directions for future research are discussed at the end. Overall, a broad and in-depth understanding of strain-tunable magnetism is very necessary, which will further drive the development of spintronics, straintronics, and flexible electronics.

## 1. Introduction

Since Geim et al. [1] successfully peeled off stable monolayer graphene in 2004, 2D materials have gradually entered the vision of scientific researchers. While pristine graphene is diamagnetic, introducing defects and strains is an effective way to obtain long-range magnetic ordering [2,3,4,5,6,7,8]. Very recently, ferromagnetism (FM) has also been found in multilayer graphene [9], graphene nanoribbons [10], graphene open-shell nanostructures [11], twisted bilayer graphene [12,13,14,15], and graphene moiré superlattice [16]. Except for graphene, MoS_2_ [17,18,19,20,21] has also attracted extensive attention. Interestingly, many experimental studies show that the defective MoS_2_ nanostructures [18,19,22,23,24,25,26,27,28,29,30,31,32,33,34,35,36,37,38,39,40,41,42,43,44,45,46,47] also exhibit FM.

Notably, strain engineering [3,4,5,6,48,49,50,51,52,53,54,55,56,57,58,59,60,61,62,63,64,65,66,67,68,69,70,71,72] is also an effective way to mediate the magnetism of 2D materials. However, most of the previous work mainly focused on theoretical calculations. We have first introduced biaxial strain into the MoS_2_ film through spontaneous buckling and found that biaxial strain can enhance its room-temperature FM (RTFM) [72,73,74]. As a whole, an extensive and in-depth understanding of strain-mediated magnetism in MoS_2_ is needed, which would provide new avenues for spintronics and straintronics.

**Figure 1:**
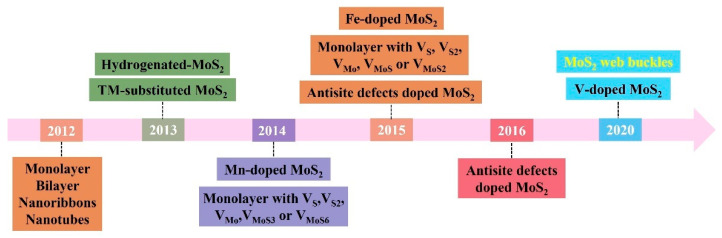
Timeline showing key developments of strain-modulated magnetism in MoS_2_. Black font represents the theoretical progress; yellow font represents the experimental progress.

Here, we will give an overview of the timeline of strain-modulated magnetism in MoS_2_ (Figure 1). We first review theoretical progress in various MoS_2_ systems, such as nanoribbons (NRs) [17,48,49,51,75,76,77,78,79,80,81,82], hydrogenated [53,67] or nitrogen-doped [70,83] systems, defective systems [27,57,60,61,63,84,85,86,87,88,89,90,91,92,93,94] and 3d transition metal ion-doped systems [55,58,68,88,95,96,97,98,99,100,101,102,103]. Then, we outline the methods of introducing strain, such as using pre-stretched substrates [104,105], bending flexible substrates [106,107,108,109,110,111,112,113,114], utilizing lattice mismatch [115,116] or thermal mismatch [72,73,113,117,118,119,120,121], alloying [122], creating buckles [72,73,104,105,114,121,123,124], using patterned substrates [125,126,127,128,129,130], bubbles [131,132,133], atomic force microscopy (AFM) tip [134,135], or piezoelectric stretching [136]. Among all the methods, creating buckles is suitable for detecting and studying the magnetism conveniently. Furthermore, we emphasize the roles played by web buckles, which induce biaxial tensile strain. Despite some progress, the study of strain-dependent MoS_2_ magnetism is still in its infancy and a few potential directions for future research are discussed at the end.

## 2. Progress in Theoretical Calculations of Strain-Mediated Magnetism

### 2.1. Nanoribbons

Similar to ZnO [80,137,138] and graphene [4,10] NRs with zigzag-terminated edges, zigzag MoS_2_ NRs also exhibit FM [17,80] independent of NRs width and thickness due to the edge atoms. In contrast, armchair NRs show non-magnetism (NM). Interestingly, introducing adatoms can enhance the net magnetic moment of armchair NRs, but the FM of zigzag NRs is inhibited by the defects caused by adatoms [75]. Because the edge atoms are passivated, their spin polarization at the Fermi level is suppressed. Furthermore, an external static electric field can also reduce the energy gap of armchair NRs [76]. In detail, this electric field will drive metal-insulator phase transformation, which modulates or even suppresses FM.

**Figure 2:**
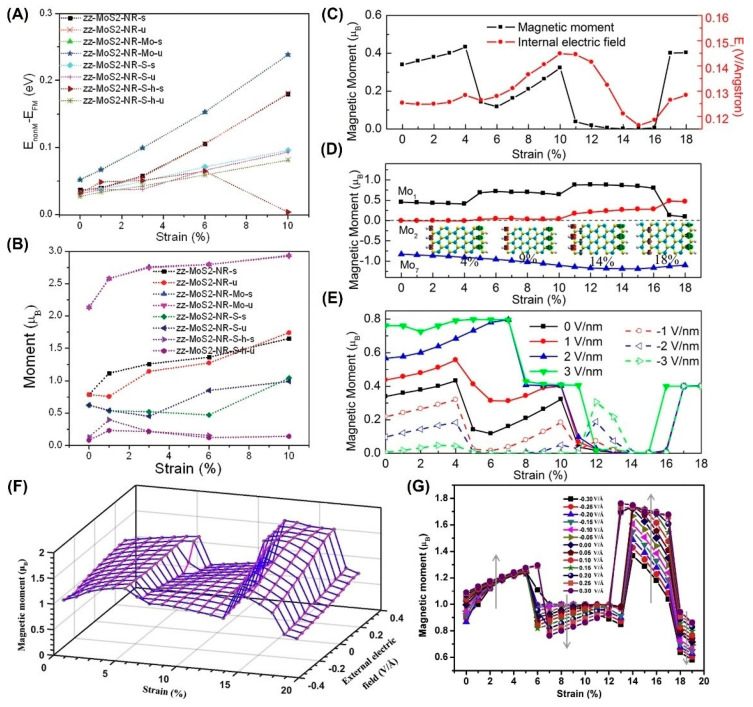
Strain-dependent magnetism in zigzag MoS_2_ NRs. (**A**,**B**) Energy difference and magnetic moment of the zigzag nanoribbons under uniaxial tensile strain along its axis. (Reprinted/adapted with permission from Ref. [51]. Copyright 2012, American Chemical Society). (**C**) Tensile strain along -dependent magnetic moment of MoS_2_ nanoribbons and the internal electric field across the edges of the ribbon (Reprinted/adapted with permission from Ref. [48]. Copyright 2012, American Chemical Society). (**D**) Magnetic moment on the edge Mo atoms versus strain. (Reprinted/adapted with permission from Ref. [48]. Copyright 2012, American Chemical Society). (**E**) Magnetic moment evolution of the strained MoS_2_ nanoribbon under electric fields. (Reprinted/adapted with permission from Ref. [48]. Copyright 2012, American Chemical Society). (**F**) 3D view of the combined effects of strain and external electric field on magnetic moment. (Reprinted/adapted with permission from Ref. [81]. Copyright 2018, Elsevier). (**G**) Magnetic moment versus the strained nanoribbon with different external electric fields (Reprinted/adapted with permission from Ref. [81]. Copyright 2018, Elsevier).

In addition, monolayer and bilayer MoS_2_ are also sensitive to tensile strain but cannot produce the long-range magnetic order in Figure 2. However, the magnetic moment in zigzag NRs is nearly doubled by 10% strain [51], as shown in Figure 2B, which may be related to the magnetic coupling from different edge atoms. As shown in Figure 2B–E, the variation is generally not monotonous [48,49,51].

Interestingly, applying tensile strain and an electric field in the zigzag direction can cause the reversible modulation of FM [48]. The applied strain is within the elastic limit of the material, which achieves the reversibility of regulation. Even for zigzag Janus MoSSe NRs, the magnetism shows a multi-stage change with the increase in strain, which is closely related to the electronic phase transition. After the electric field is applied again, the magnetism can be regulated more effectively [81].

However, this modulation is obviously different from that of the zigzag MoS_2_ NRs shown in Figure 2E–G. Indeed, the difference in local spin density distribution determines the different modulation results of zigzag MoS_2_ NRs and zigzag Janus MoSSe NRs.

### 2.2. Hydrogenated or Nitrogen-Doped Systems

Even after applying the biaxial tensile strain from about −8% to 8%, pristine monolayer and bilayer 2H-MoS_2_ [49] are NM, indicating that no spin polarizations are aligned to form FM. However, other dichalcogenides materials, such as pristine VS_2_ and VSe_2_, exhibit FM [50], and the FM will increase rapidly when the strain increases from −5% to 5%. Metallic materials such as pristine 1T-MoS_2_ [33,37,41,67,139,140], VS_2_ [50,141,142] and VSe_2_ [50,141,142,143,144,145] monolayer are more likely to form spontaneous magnetization.

In addition, the contribution of V atoms to magnetism is much greater than that of S or Se atoms [50]. In contrast, unstrained NbS_2_ and NbSe_2_ monolayers [52] are NM but can produce between 0.50 and 0.61 µ_B_ per unit cell after applying 5% biaxial tensile strain. This novel magnetic behavior of NbS_2_ and NbSe_2_ monolayers is related not only to the bond length increased by strain but also to the metallic properties.

In fact, the self-exchange of populations between 4d orbitals of Nb atoms can lead to spin splitting [52,56]. Overall, V or Nb 4d states contribute mainly to the metallic state near the Fermi energy level [50,52,56]. By applying strain, the Curie temperature of the materials may be raised above room temperature [52], which will accelerate the spintronic application of 2D magnetic materials. However, MoS_2_, WS_2_, MoSe_2_, and WSe_2_ have no intrinsic magnetism [52] due to their characteristic band structures.

**Figure 3:**
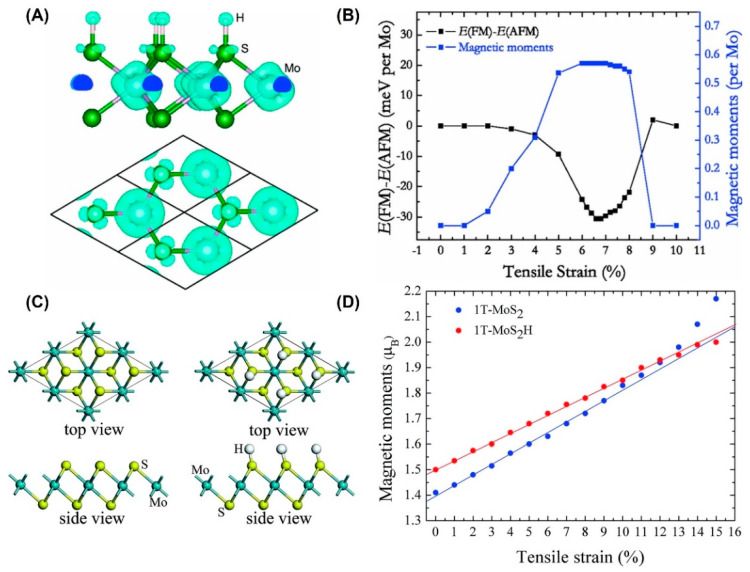
Strain-dependent magnetism in hydrogenated monolayer MoS_2_. (**A**) Contour plots of the spin density of hydrogenated monolayer 2H-MoS_2_ under the biaxial tensile strain of 6%. (Reprinted/adapted with permission from Ref. [53]. Copyright 2013, American Physical Society). (**B**) Energy difference per Mo atom for 2H-MoS_2_H and the magnetic moment of Mo *d* orbitals per Mo as a function of strain (Reprinted/adapted with permission from Ref. [53]. Copyright 2013, American Physical Society). (**C**) Monolayer 1T-MoS_2_ model without and with hydrogen adsorption (Reprinted/adapted from Ref. [67] with permission from the Royal Society of Chemistry). (**D**) The function of magnetic moments of Mo atom in 1T-MoS_2_ as tensile strain (Reprinted/adapted from Ref. [67] with permission from the Royal Society of Chemistry). Note that: The 2H phase structure with space group Pm2 has hexagonal symmetry and the primitive unit cell of the single-layer has three atoms. The S atom is with trigonal prismatic coordination around Mo atoms; The 1T phase is also with hexagonal symmetry and the primitive unit cell of the single-layer has three atoms. In the 1T phase with space group Pm1, the S atom is with octahedral coordination around Mo atoms.

Hydrogen atoms [53,67] can modify the electronic structure of pristine 2H-MoS_2_, but cannot produce spontaneous magnetism under <3% tensile strain [53], as shown in Figure 3A. With the increase in biaxial tensile strain, the magnetic moment and stability will be enhanced, as shown in Figure 3B. When the strain reaches 6.6%, the supercell obtains the most stable FM state, and the magnetic moment reaches 0.57 µ_B_ per unit cell. In addition, its Curie temperature (Tc, ~232 K) is much higher than that of the transition metal (TM)-doped system (Tc, ~40 K) [84].

However, 1T-MoS_2_ and 1T-MoS_2_H show FM behaviors, as shown in Figure 3C. Unlike 2H-MoS_2_, the relationship between magnetic moments and strain is linear, as shown in Figure 3D [67]. The crystal field makes a great contribution to the magnetism of the system.

Similarly, the biaxial tensile strain can also modulate the magnetism of nitrogen-doped 2H-MoS_2_ [70]. When the strain gradually increases to 17.09%, a single nitrogen doping structure (NMo_16_S_31_) shows different magnetic phases. However, the magnetic moment of a dense nitrogen doping structure (NMo_4_S_7_) steps from 0 up to 1 µ_B_ under 14% strain. In detail, unpaired electrons doped with nitrogen atoms will induce magnetic order. When the doped nitrogen atoms are too dense, the magnetic order will be weakened. However, the biaxial tensile strain has a good modulation effect on these two structures.

### 2.3. Defective Strained Systems

Inspired by the magnetism caused by conductive electrons in defective graphene. Many research groups tried to introduce single vacancies into the MoS_2_ monolayer [57,94]. Experimentally, atomic single vacancies [91] (V_Mo_: mono-molybdenum vacancy; V_S_: mono-sulfur vacancy; V_2S:_ disulfur vacancy), vacancy complexes (V_MoS3_: vacancy complex of Mo and nearby three sulfur; V_MoS6_: vacancy complex of Mo nearby three disulfur pairs) and antisite defects [61,63] (S_2Mo_: an S2 column substituting a Mo atom; Mo_S_: a Mo atom substituting an S column; Mo_2S_: a Mo atom substituting an S2 column) have been observed in CVD (chemical vapor deposition)-grown MoS_2_ monolayer by atomic-resolution annular dark field (ADF) imaging on an aberration-corrected scanning transmission electron microscope (STEM) [86]. Through first-principles calculations shown in Table 1, it is found that pristine [60,94] and single vacancy [57,60,94]-MoS_2_ monolayer are NM. Notably, when 19% biaxial tensile strain is applied to the pristine MoS_2_ monolayer, 4 × 4 supercells produce a magnetic moment of 5 µ_B_. However, the uniaxial strain cannot cause a magnetic phase transition regardless of the applied direction.

**Figure 4:**
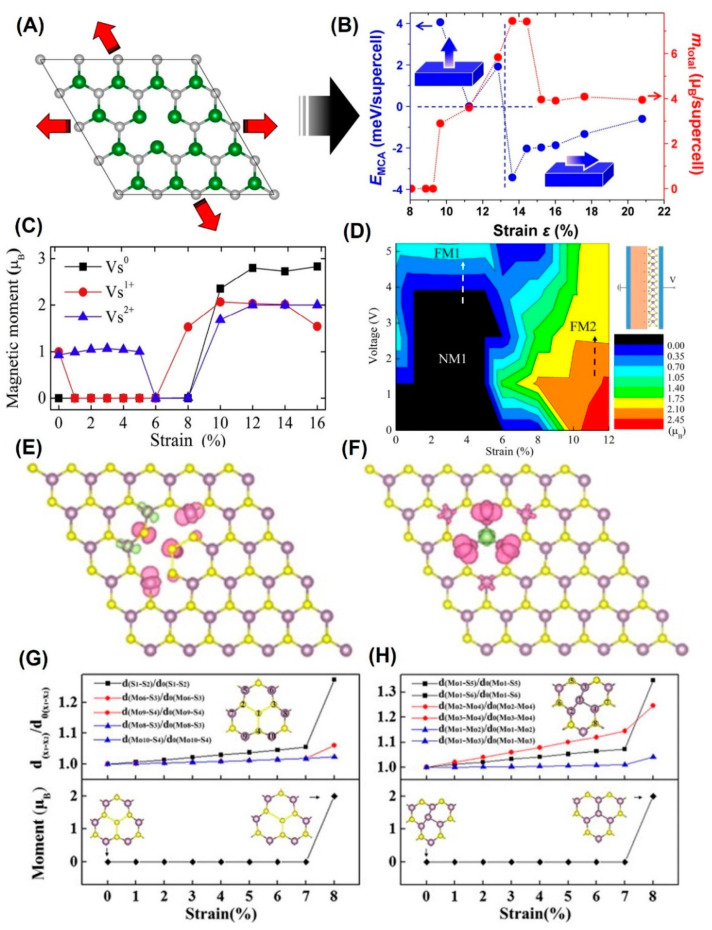
(**A**) Schematic illustration of MoS_2_ ML with V_2S_ under biaxial tensile strain (Reprinted/adapted with permission from Ref. [60]. Copyright 2013, American Physical Society). (**B**) E_MCA_ and magnetic moment vs. applied tensile strain. (Reprinted/adapted with permission from Ref. [60]. Copyright 2013, American Physical Society). (**C**) The magnetic moments of V_S_-MoS_2_ under strain with charge state q = 0, 1, 2. (Reprinted/adapted with permission from Ref. [91]. Copyright 2018, Elsevier). (**D**) The magnetic phase diagram of V_S_-MoS_2_ driven by strain and voltage with a capacitor structure. (Reprinted/adapted with permission from Ref. [91]. Copyright 2018, Elsevier). (**E**,**F**) Spin density distributions of MoS_2_ systems with S_2Mo_ and Mo_S2_ under 8% strains. (Reprinted/adapted with permission from Ref. [61]. Copyright 2015, Elsevier). (**G**,**H**) The evolutions of magnetic moments of the supercell and the parameter d/c_0_ with the strain for the V_S_ and V_2S_. (Reprinted/adapted with permission from Ref. [61]. Copyright 2015, Elsevier).

Interestingly, unstrained MoS_2_ monolayers with V_Mo_ [60,94], V_S_ [57,60], V_2S_ [57,60,94], V_MoS_ [60], V_MoS3_ [57], S_2Mo_ [61] and Mo_S2_ [61] are NM, as shown in Figure 4, while unstrained MoS_2_ monolayers with V_MoS2_ [46,60,146], V_MoS6_ [57] and Mo_S_ [63] are magnetic. In detail, the charge transfer and Mo atoms around the defects contribute mainly to magnetism. Furthermore, spin reorientation and the largest magnetic moment occur in the V_2S_-MoS_2_ monolayer [60], as shown in Figure 4A,B, which is related to magneto-crystalline anisotropy. With the increase in the tensile strain, FM-NM-FM phase transformation has been observed in V_S_-MoS_2_. Li et al. [91] have also drawn the magnetic phase diagram caused by strain and external electric field, as shown in Figure 4C,D. After applying strain, the charge sulfur vacancy defect shows rich magnetic responses.

Since Zhou et al. [86] and Jin et al. [147] found the antisite defects in the MoS_2_ monolayer by STEM imaging in 2013, researchers have been trying to understand their magnetic characteristics in Figure 4E–H. In detail, the defect is an intrinsic structural defect. After applying 8% biaxial tensile strain, the system will produce long-range magnetic order [61]. Overall, the spin density is mainly distributed in the sulfur atom and its nearest or second neighbor, the Mo atom. However, the antisite-doped monolayer exhibits a high spin state under the biaxial strain from −7% to 4%. With the further increase in tensile strain, magnetism will vanish. The position of the antisite atom is related to the magnetism of the system. In addition, it is found that strained V_S_ can greatly improve the hydrogen evolution activity of MoS_2_ basal planes [148]. The sulfur vacancy will become a new active site and tune the adsorption-free energy of the hydrogen atom.

### 2.4. 3d Transition Metal (TM) Ion-Doped Systems

Doping engineering [149,150,151,152,153,154,155,156,157] is a traditional way to control the properties of materials, especially for 2D materials. Recently, it has been confirmed experimentally [149,150,158,159,160,161,162] that 3d TM doping can induce ferromagnetism in nonmagnetic MoS_2_, which can be combined with strain engineering to tune the magnetism, as shown in Table 2.

Interestingly, TM-doped systems show different magnetic responses. Except for V, Cr, Ti, and Sc atoms [88], the TM-doped systems without strain are nonmagnetic, and no matter how much biaxial strain is applied, there will be no long-range magnetic order. Arguably, Ma et al. [100] reported that V-doped monolayer MoS_2_ exhibits magnetic half-metal at zero strain. After 2% compressive strain or 3% tensile strain is applied, the system will change from an FM state to an antiferromagnetic state.

Notably, the magnetic properties of Co/Ni/Cu/Zn-doped molybdenum disulfide show nonlinear changes with strain. After applying 20% compressive strain, the system is nonmagnetic. When the applied tensile strain reaches a specific value, the system will obtain a high spin state (5 µ_B_ for the Co-doped; 5 µ_B_ for the Ni-doped; 5 µ_B_ for the Cu-doped; 3 µ_B_ for the Zn-doped). However, the magnetic moment will reduce to 0 under 20% tensile strain, except for the Co-doped system (3 µ_B_).

The linear monotonicity of magnetism with strain has also been found in Mn-doped and Fe-doped MoS_2_ systems, which is similar to those of 1T-MoS_2_ and 1T-MoS_2_H. In detail, the systems are NM under a 20% compressive strain. Appling 20% tensile strain, the systems have obtained high spin states (2.8 µ_B_ for Mn-doped; 4.3 µ_B_ for Fe-doped).

In general, strain engineering is an effective method to control the magnetism of the TM-doped molybdenum disulfide system.

## 3. Experimental Progress of Strain-Mediated Magnetism

### 3.1. Methods of Appling Strain

Since the experiments revealed that 2D materials can withstand up to 20% strain, strain-modulated magnetism has gradually become an emerging research field. However, it is difficult to apply strain directly in suspended 2D materials in Table 3.

In 2013, Andres et al. [104] created wrinkles in few-layer MoS_2_ by pre-stretching the gel-film substrate, resulting in uniaxial tensile strain up to 2.5%. In the same year, uniaxial tensile strain (0–2.2%) was also applied in the MoS_2_/polycarbonate system by using four-point bending equipment [106]. Since then, many research groups have tried to apply strain through a variety of flexible substrates, including polymers [107], polyethyleneterephthalate (PET) [108,109], polyvinyl alcohol (PVA) [110], polyimide (PI) [111,112] and polydimethylsiloxane (PDMS) [113,114].

In addition, the researchers have found that the intrinsic tensile strain (0.15–1.37%) was also introduced in CVD grown-monolayer MoS_2_ [113,115,117,118,119,122]. This intrinsic tensile strain is caused by the mismatch of thermal expansion coefficients [72,73,113,115,116,117,121]. Interestingly, whether through flexible substrate [104] or thermal mismatch [72,73], the strain state of MoS_2_ materials can be further mediated by creating buckles [72,73,104,105,114,121,123,124].

Recently, it has also been experimentally found that the strain can be introduced into the materials through patterned substrates such as holey Si_3_N_4_ [125], rippled Si/SiO_2_ [126], SiO_2_ nanocones [127], SiO_2_ nanopillars [128], pyramid/cones Al_2_O_3_ [129], ZnO nanorods arrays [130], nanodots arrays, and so on. During the transfer of MoS_2_ samples, bubbles [131,132,133] are often formed to introduce large strains into the samples. Notably, most of the methods required additional equipment to provide external stimulation, such as an AFM tip [134,135], an electromechanical device [74], or a focused laser beam [136]. Because scanning superconducting quantum interference device (SQUID) needs to be conducted in a cryogenic temperature and vibration environment, it is difficult to detect the strained material system. So far, material systems that can spontaneously form buckles [72,73,74] are more suitable for magnetic study.

### 3.2. Spontaneous Formation of Web Buckles

Spontaneous buckling [163,164] is frequently observed in the film system of traditional materials. When the residual strain in the film reaches its critical value, it will drive the film to delamination from the substrate and from spontaneous wrinkles [72,73,74]. Interfacial adhesion [73,165] is one of the key factors in determining whether buckling is formed or not. Relatively low adhesion is conducive to the formation and propagation of buckles. Because there is no hanging bond on the surface of 2D materials such as MoS_2_, the van der Waals (vdW) force is the interaction between the material and the substrate, and its interface adhesion is relatively low. Since then, MoS_2_ films are very likely to become the perfect platform for understanding the phenomena of spontaneous buckling [73].

**Figure 5:**
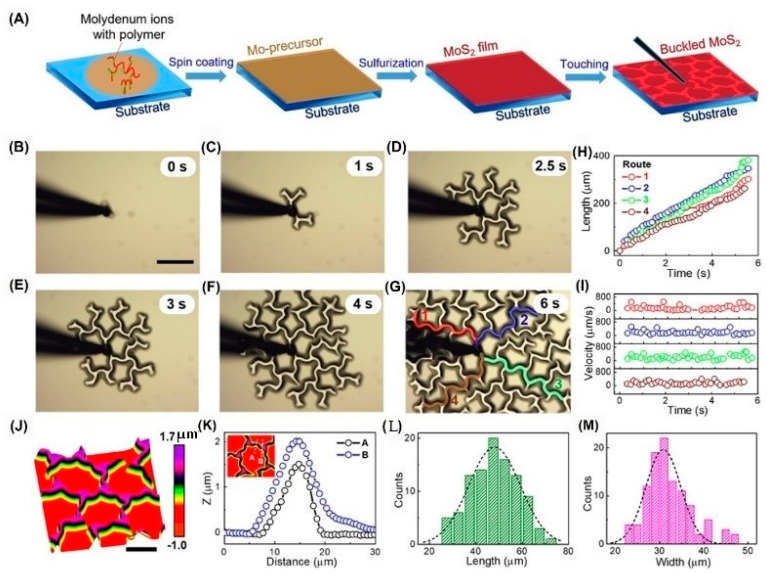
Formation of large-area web buckles. (**A**) Schematic illustration of the growth of a MoS_2_ thin film with PAD and the triggering of buckles by a probe touching. (**B**–**G**) In situ observation of large area web buckles formed on an as-grown MoS_2_ thin film with a thickness of 370 nm. Scale bar, 100 μm. (**H**,**I**) Propagating distances and velocities of buckles along four different branches as labeled in (**G**), as a function of time, respectively. (**J**) AFM 3D topography of a buckled MoS_2_ thin film with a thickness of 230 nm. Scale bar, 20 μm. (**K**) Two height -profile lines crossing the middle of a telephone cord (line A) and a node position (line B) as shown in the inset. (**L**,**M**) Statistical histograms of lengths and widths of buckles. Reprinted/adapted with permission from Ref. [73]. Copyright 2019, American Chemical Society.

Recently, our group prepared ultra-smooth MoS_2_ films [72,73] by polymer-assisted deposition (PAD), as shown in Figure 5. When the thickness of the film is about 400 nm, its roughness is about 1 nm. In the laboratory environment, MoS_2_ films will also spontaneously form buckles due to external disturbance. Inspired by this experimental observation, we have used a tungsten probe close to the touch film to apply a point load. Once the probe touches the film, web buckles will be formed and further spread to the whole film surface. The formed large-area film with web buckles is very suitable for the SQUID test. Surprisingly, there is no obvious damage to the web buckle’s structure after the magnetic test.

### 3.3. Web Buckle-Mediated RTFM

Strain engineering [6,49,51,52,56,57,58,61,72,73,166] is a straightforward way to mediate the magnetism of MoS_2_. However, most of the previous work [48,49,50,51,52,53,55,57,58,60,61,63,67,68,70,81,88,90,91,94,97,98,100] mainly focused on theoretical calculations. In the experiment, it was very difficult to apply biaxial strain directly to 2D materials. In order to clarify the strain-mediated FM in MoS_2_, the following problems must be solved: (1) how to quantitatively determine the strain in the system experimentally; (2) how to select two suitable strain states to study their ferromagnetism; (3) how to measure ferromagnetism in different zones of web buckles.

**Figure 6:**
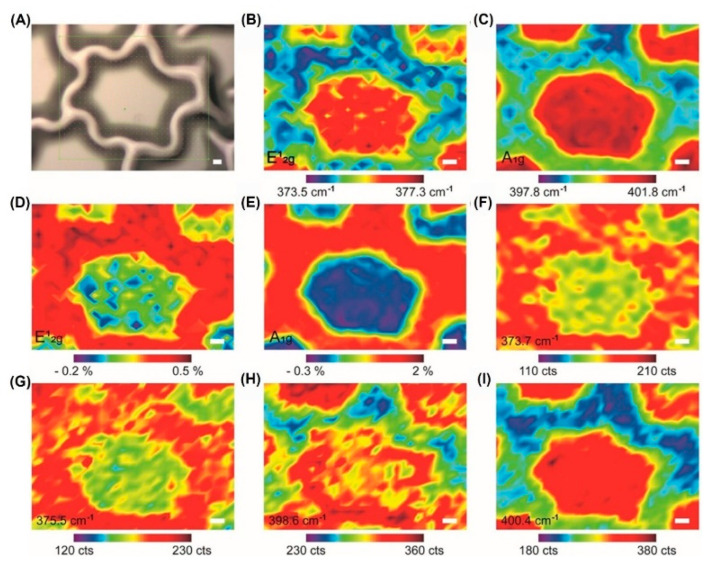
Native strain variations in MoS_2_ web buckles. (**A**) Optical image of MoS_2_ web buckles. (**B**,**C**) Raman position mapping in the E^1^_2g_ and A_1g_ modes. (**D**,**E**) Strain mapping of MoS_2_ web buckles is estimated by the response of Raman-active modes to the applied biaxial strain for single-layer MoS_2_. (**F**–**I**) Raman intensity mapping in E^1^_2g_ (between 373.7 cm^−1^ and 375.5 cm^−1^) and A_1g_ (between 398.6 cm^−1^ and 400.4 cm^−1^). Scale bars: 5 μm. (Reprinted/adapted with permission from Ref. [72]. Copyright 2020, American Institute of Physics).

Since Ferrari et al. [167] successfully measured the uniaxial and biaxial strain in graphene samples in 2009, Raman spectroscopy has become a powerful tool to characterize the strain deformation of two-dimensional materials. Soon after 2013, the strain-tunable energy gap was studied in mono-, bi-, and tri-layer MoS_2_ [104,106,136,168,169]. Notably, Yagmurcukardes et al. [170] studied how the strain modulated the Raman characteristics of single-layer materials by first-principle calculation. Therefore, we used Raman spectroscopy to quantify the strain in web buckles (Figure 6) [72,73,74]. In detail, it is estimated by Raman mapping that about 68% of the region in the flat film has strain variations.

**Figure 7:**
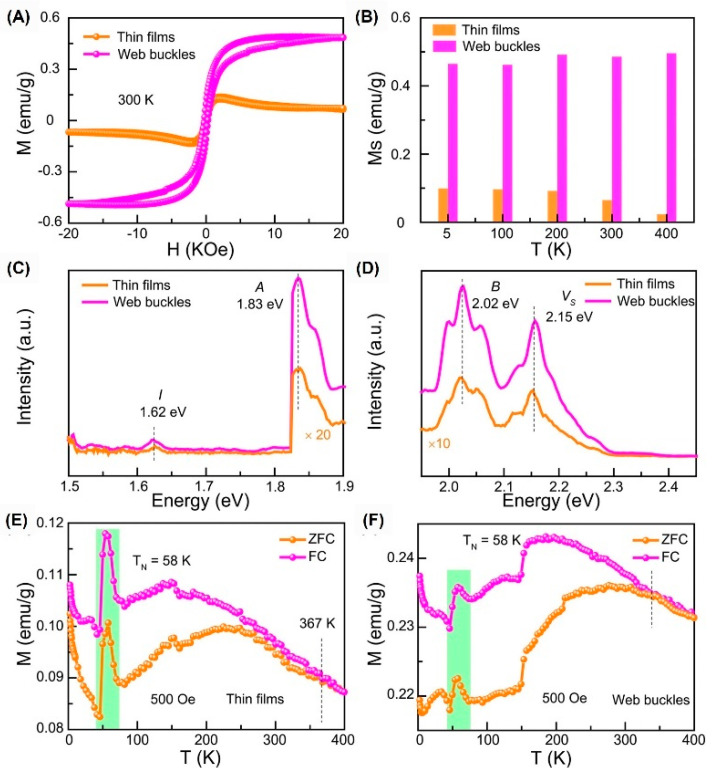
Ferromagnetism of MoS_2_ thin films and web buckles. M-H curves (**A**), M_s_–T (**B**), FL excitation spectra (**C**), and FL emission spectra (**D**) of MoS_2_ thin films and web buckles. (**E**,**F**) M_s_–T of MoS_2_ thin films and web buckles. (Reprinted/adapted with permission from Ref. [72]. Copyright 2020, American Institute of Physics).

In order to clarify the strain-dependent ferromagnetism, we selected flat films and buckled films to test, as shown in Figure 7. After buckling, the saturation magnetization at 300 K increases to 7.5 times that before buckling. This is because the biaxial tensile strain induced by web buckles produces the generation of more defects such as V_S_. The enhancement of magnetism may be related to the decrease in compressive strain and the increase in defects.

So far, we cannot distinguish the magnetism from different buckled areas. Although traditional magnetic force microscopy can be obtained, we believe that there are too many impurity signals to identify the information in the samples. Hopefully, the newly emerging magnetic imaging technologies will provide technical support for further research.

## 4. Conclusions and Outlook

In this review, we have summarized the recent developments in strain-dependent magnetism in MoS_2_. First, we reviewed the progress of the theoretical study. Then, we compared the experimental methods of introducing strain and their effects on the ferromagnetism. We emphasized the roles played by web buckles since they could induce biaxial tensile strain conveniently for further tests, including magnetic measurements. Obviously, despite some progress, the study of strain-dependent MoS_2_ magnetism is still in its infancy.

Although RTFM has been enhanced experimentally by biaxial strain [72] induced by web buckles, the magnetism contributions from different zones cannot be distinguished experimentally. Since most conventional magnetic probes [171] require the sample area to be at the millimeter level, magnetic testing of the micron wrinkled area is a great challenge. Very recently, magnetic imaging techniques have emerged as important tools for investigating 2D materials, such as magnetic force microscopy (MFM) [172,173,174,175,176,177,178], SQUID [179,180], magneto-optical Kerr effect (MOKE) [181,182] and scanning nitrogen-vacancy center microscopy (SNVM) [183,184,185,186,187]. These techniques make it possible to detect the magnetism of the wrinkled area.

Since the modulation effect of uniaxial strain on the properties of materials is weaker than that of biaxial strain, whether the RTFM of molybdenum disulfide can be regulated by uniaxial strain has always been a mystery, which is worthy of further exploration. In addition, the substrates commonly used in experiments are isotropic, so it is relatively easy to introduce isotropic strain (such as biaxial strain) into 2D materials. Recently, anisotropic substrates such as *m*-quartz [121,188] have been used in experiments, which provides a new idea for introducing uniaxial strain into MoS_2_. We believe that the regulation of uniaxial strain on FM can be explained clearly by combining nanoscale magnetic detection instruments.

Overall, an extensive and in-depth understanding of strain-mediated magnetism in MoS_2_ is needed, which would provide new avenues for spintronics [189,190,191,192,193,194,195,196,197] and straintronics [198,199,200,201,202].

## Figures and Tables

**Table 1:** Strain-dependent magnetism of single-layer MoS_2_ with various defects.

System	Supercell Size	Maximum Strain	Magnetic Moment	Remarks
Pristine [94]	4 × 4	11%	0 µ_B_	NM (0–11%), biaxial
Pristine [60]	4 × 4	20%	5 µ_B_ (19%)	NM (0–20%), biaxial
Pristine [60]	$4\;\times \;2\sqrt{3}$	20%	0 µ_B_	NM (0–20%), x-axis
Pristine [60]	$4\;\times \;2\sqrt{3}$	20%	0 µ_B_	NM (0–20%), y-axis
V_Mo_ [94]	4 × 4	11%	>2 µ_B_ (7–11%)	NM (<7%), biaxial
V_Mo_ [60]	4 × 4	20%	2.02 µ_B_ (14.5%)	NM (<6.5%), biaxial
V_Mo_ [60]	$4\;\times \;2\sqrt{3}$	20%	2.02 µ_B_ (7.5–20%)	NM (0–7.5%), x-axis
V_Mo_ [60]	$4\;\times \;2\sqrt{3}$	20%	2.02 µ_B_ (7.5–20%)	NM (0–7.5%), y-axis
V_S_ [57]	6 × 6	10%	2.0 µ_B_ (9%)	NM (<9%), biaxial
V_S_ [60]	4 × 4	20%	4.07 µ_B_ (14.5%)	NM (<8%), biaxial
V_S_ [60]	$4\;\times \;2\sqrt{3}$	20%	~2.07 µ_B_ (20%)	NM (0–15%), x-axis
V_S_ [60]	$4\;\times \;2\sqrt{3}$	20%	~2.0 µ_B_ (15%)	NM (0–10%), y-axis
V_S_^0^ [91]	4 × 4	16%	~3 µB (12%)	NM (0–10%), biaxial
V_S_^1+^ [91]	4 × 4	16%	2.0 µB (12%)	NM (0–6%), biaxial
V_S_^2+^ [91]	4 × 4	16%	2.0 µB (12%)	NM (0%;6–8%), biaxial
V_2S_ [94]	4 × 4	11%	>2 µ_B_ (>10%)	NM (<10%), biaxial
V_2S_ [57]	6 × 6	10%	5.5 µ_B_ (9%)	NM (<9%), biaxial
V_2S_ [60]	4 × 4	22%	7.45 µ_B_ (13.5%)	NM (<9.5%), biaxial
V_2S_ [60]	$4\;\times \;2\sqrt{3}$	20%	~3.0 µ_B_ (20%)	NM (0–15%), x-axis
V_2S_ [60]	$4\;\times \;2\sqrt{3}$	20%	~1.0 µ_B_ (20%)	NM (0–15%), y-axis
V_MoS_ [60]	4 × 4	20%	4.04 µ_B_ (13%)	NM (<5.5%), biaxial
V_MoS_ [60]	$4\;\times \;2\sqrt{3}$	20%	~1.7 µ_B_ (10%);	NM (<10%, 15%), x-axis
V_MoS_ [60]	$4\;\times \;2\sqrt{3}$	20%	0 µ_B_	NM (0–20%), y-axis
V_MoS__2_ [60]	4 × 4	20%	~5.9 µ_B_ (20%)	~2 µ_B_ (<5%), biaxial
V_MoS__2_ [60]	$4\;\times \;2\sqrt{3}$	20%	0 µ_B_	NM (>4%), x-axis
V_MoS__2_ [60]	$4\;\times \;2\sqrt{3}$	20%	~2 µ_B_ (0–20%)	y-axis
V_MoS__3_ [57]	6 × 6	10%	4.0 µ_B_ (10%)	NM (<10%), biaxial
V_MoS__6_ [57]	6 × 6	−12%	12.0 µ_B_ (9%)	NM (−12%), biaxial
S_2Mo_ [61]	6 × 6	8%	2.0 µB (8%)	NM (<8%), biaxial
Mo_S2_ [61]	6 × 6	8%	2.0 µB (8%)	NM (<8%), biaxial
Mo_S_ [63]	4 × 4	7%	2.0 µB (−7–4%)	NM (5–7%), biaxial

**Table 2:** Strain-dependent magnetism of TM-doped single-layer MoS_2_.

Dopant	Supercell Size	Maximum Strain	Magnetic Moment/µ_B_	Remarks
V [88]	5 × 5	20%	0 µ_B_ (−20–20%)	
V [100]	4 × 4	5%	0.81 µ_B_ (0%)	AFM (3% or −2%)
Mn [88]	5 × 5	20%	1.0 µ_B_ (0%)	0 (−20%); 2.8 µ_B_ (20%)
Mn [55]	4 × 4	6%	1.0 (1%)	3.0 µ_B_ (6%), biaxial
Mn [98]	4 × 4	−10%	1.0 (−10–9%)	be almost independent on the size of supercell, no matter under a tensile or compressive strain
Mn [98]	5 × 5	−10%	1.0 (−10–9%)	
Mn [98]	6 × 6	−10%	1.0 (−10–9%)	
Mn [98]	Unit cell	9%	1.0 µ_B_ (0–3%)	3.0 µ_B_ (4–9%), biaxial
Fe [88]	5 × 5	20%	2.0 µ_B_ (0%)	0 (−20%); 4.2 µ_B_ (20%)
Fe [58]	4 × 4	6%	2.04 µ_B_ (0%)	4.0 µ_B_ (3.5–6%), spin reorientation
Fe [68]	Unit cell	9%	2.0 µ_B_ (0–5%)	4.0 µ_B_ (6–9%), biaxial
Co [88]	5 × 5	20%	5.0 µ_B_ (15%)	0 (−20%); 3.3 µ_B_ (20%)
Co [68]	Unit cell	9%	3.0 µ_B_ (0–7%)	3.4 µ_B_ (8%), biaxial
Ni [88]	5 × 5	20%	5.0 µ_B_ (10%)	0 (−20%); 2.0 µ_B_ (20%)
Ni [68]	Unit cell	9%	4.0 µ_B_ (0–8%)	3.7 µ_B_ (9%), biaxial
Cu [88]	5 × 5	20%	5.0 µ_B_ (0%)	0 (−20%); 0 µ_B_ (20%)
Zn [88]	5 × 5	20%	3.0 µ_B_ (10%)	0 (−20%); 0 µ_B_ (20%)
Cr [88]	5 × 5	20%	0 µ_B_ (−20–20%)	
Ti [88]	5 × 5	20%	0 µ_B_ (−20–20%)	
Sc [88]	5 × 5	20%	0 µ_B_ (−20–20%)	

**Table 3:** The range and types in strained MoS_2_ systems by different inducing methods. HOPG: Highly oriented pyrolytic graphite; PMN-PT: [Pb(Mg_1/3_Nb_2/3_)O_3_]_0_._7_-[PbTiO_3_]_0_._3_. δ_mem_: the deflection of the membrane.

Methods	Substrates	Layers	Ranges	Remarks
Pre-stretches substrate	Gel-film [104]	3−5 L	0.2–2.5%	Uniaxial tensile
Pre-stretches substrate	PDMS [105]	2–10 L	20% (PDMS)	Uniaxial
Flexible substrate	Polycarbonate [106]	1−2 L	0−2.2%	Uniaxial tensile
Flexible substrate	Polymer [107]	1, few	0–0.8%	Uniaxial tensile
Flexible substrate	Ag-coated PET [108]	20–80 nm	0–0.02%	Uniaxial tensile
Flexible substrate	PET [109]	1 L	−0.7–0.7%	Uniaxial
Flexible substrate	PVA [110]	1 L	0–1.49%	Uniaxial tensile
Flexible substrate	Polyimide [111]	1–2 L	0–0.32%	Uniaxial tensile
Flexible substrate	Polyimide [112]	2 L	0–1.19%	biaxial
Flexible substrate	PDMS [113]	1 L	0–4.8%	Uniaxial tensile
Flexible substrate	PDMS [114]	2–10 L	~2.2%	Uniaxial tensile
Lattice mismatch	Si/SiO_2_ [115]	1 L	~1.24%	Intrinsic tensile
Lattice mismatch	HOPG [116]	1 L	~1.76%	Anisotropic tensile
Thermal mismatch	Si/SiO_2_ [113]	1 L	~1.0%	Intrinsic tensile
Thermal mismatch	Si/SiO_2_ [117]	1 L	0.4%; 0.6%	Intrinsic tensile
Thermal mismatch	Si/SiO_2_ [118]	1 L	~0.76%	Intrinsic tensile
Thermal mismatch	Si/SiO_2_ [119]	2 L	~0.34%;	Intrinsic compressive
Thermal mismatch	Sapphire [117]	1 L	0.15%; 0.2%	Intrinsic tensile
Thermal mismatch	*h*-BN [117]	1 L	~0.8%; ~0.2%	Intrinsic tensile
Thermal mismatch	Mica [117]	1 L	~0.8%; ~0.2%	Intrinsic tensile
Thermal mismatch	PDMS [120]	1 L	<−0.2%	Biaxial compressive
Thermal mismatch	Al_2_O_3_ [72,73]	~60 nm	−0.29–−0.45%	Biaxial compressive
Thermal mismatch	m-quartz [121]	1 L	~−0.776%	Uniaxial compressive
Alloying	MoS_2x_Se_2(1__−__x)_ [122]	1 L	<4%	Biaxial tensile
Creating buckles	Gel-film [104]	3−5 L	0.2–2.5%	Uniaxial tensile
Creating buckles	PDMS [114]	2–10 L	~2.2%	Uniaxial
Creating buckles	PDMS [105]	2–10 L	~1–~2%	Uniaxial compressive
Creating buckles	Al_2_O_3_ [72,73]	~60 nm	−0.45–1.7%	Biaxial
Creating buckles	m-quartz [121]	1 L	0.14–1.58%	Uniaxial tensile
Creating buckles	Au films [123]	1 L	−1.16–2.04%	Uniaxial
Creating buckles	Si/SiO_2_ [124]	10–21 nm	0.32–1.11%	Uniaxial tensile
Patterned substrate	Holey Si_3_N_4_ [125]	2 L	~1.8%	Biaxial tensile
Patterned substrate	Rippled Si/SiO_2_ [126]	4 L	~0.5%	Uniaxial tensile
Patterned substrate	SiO_2_ nanocones [127]	1 L	~0.565%	Biaxial tensile
Patterned substrate	SiO_2_ nanopillars [128]	1 L	~2%	Uniaxial tensile
Patterned substrate	Cone-Al_2_O_3_ [129]	2 L	~0.04%	Tensile/compressive
Patterned substrate	Pyramid-Al_2_O_3_[129]	2 L	~0.05%	Tensile/compressive
Patterned substrate	ZnO rods [130]	1 L	0–~0.6%	Periodic biaxial
Bubbles	PDMS [131]	1, few	2.9−3.5%	Biaxial tensile
Bubbles	*h*-BN [132]	1 L	~2%	Gradient tensile
Bubbles	Si/SiO_2_ cavity [133]	multi-	−0.8–1.5%	Biaxial, >5.6%
AFM tip	Si/SiO_2_ [134]	1–3 L	δ_mem_: ~33 nm	Isotropic
AFM tip	Si/SiO_2_ [135]	1 L	4.7 × 10^−5^ F	Isotropic
Piezoelectric substrate	PMN-PT [136]	3 L	0–0.2%	Biaxial compressive

## Data Availability

Not applicable.
